# Untargeted Metabolomics Approach Reveals Differences in Host Plant Chemistry Before and After Infestation With Different Pea Aphid Host Races

**DOI:** 10.3389/fpls.2019.00188

**Published:** 2019-02-28

**Authors:** Carlos Sanchez-Arcos, Marco Kai, Aleš Svatoš, Jonathan Gershenzon, Grit Kunert

**Affiliations:** ^1^Department of Biochemistry, Max-Planck Institute for Chemical Ecology, Jena, Germany; ^2^Research Group Mass Spectrometry/Proteomics, Max-Planck Institute for Chemical Ecology, Jena, Germany

**Keywords:** *Acyrthosiphon pisum*, pea aphid host races, legume metabolome, saponins, flavonoids, *Medicago sativa*, *Trifolium pratense*, *Pisum sativum*

## Abstract

The pea aphid (*Acyrthosiphon pisum*), a phloem-sucking insect, has undergone a rapid radiation together with the domestication and anthropogenic range expansion of several of its legume host plants. This insect species is a complex of at least 15 genetically different host races that can all develop on the universal host plant *Vicia faba*. However, each host race is specialized on a particular plant species, such as *Medicago sativa*, *Trifolium pratense*, or *Pisum sativum*, which makes it an attractive model insect to study ecological speciation. Previous work revealed that pea aphid host plants produce a specific phytohormone profile depending on the host plant – host race combination. Native aphid races induce lower defense hormone levels in their host plant than non-native pea aphid races. Whether these changes in hormone levels also lead to changes in other metabolites is still unknown. We used a mass spectrometry-based untargeted metabolomic approach to identify plant chemical compounds that vary among different host plant-host race combinations and might therefore, be involved in pea aphid host race specialization. We found significant differences among the metabolic fingerprints of the four legume species studied prior to aphid infestation, which correlated with aphid performance. After infestation, the metabolic profiles of *M. sativa* and *T. pratense* plants infested with their respective native aphid host race were consistently different from profiles after infestation with non-native host races and from uninfested control plants. The metabolic profiles of *P. sativum* plants infested with their native aphid host race were also different from plants infested with non-native host races, but not different from uninfested control plants. The compounds responsible for these differences were putatively identified as flavonoids, saponins, non-proteinogenic amino acids and peptides among others. As members of these compound classes are known for their activity against insects and aphids in particular, they may be responsible for the differential performance of host races on native vs. non-native host plants. We conclude that the untargeted metabolomic approach is suitable to identify candidate compounds involved in the specificity of pea aphid – host plant interactions.

## Introduction

Insects are the most diverse group of eukaryotic species on earth (Stork, 1993), and herbivorous species constitute a major group of insects (Strong et al., 1984; Mitter et al., 1988). Many herbivorous insect species are specialized on certain plant species (Bernays and Graham, 1988). Even within a single insect species, specialization can occur with different populations feeding preferentially on different plant taxa (Drès and Mallet, 2002). Adaptation to multiple plant species can lead to the formation of host races or biotypes within a species of an insect herbivore and might therefore play an important role in insect speciation. Nevertheless, the mechanisms behind these adaptations are hardly understood so far. Why is a certain host race able to feed on one plant but not another one? To some extent, differences in the constitutive chemical profiles of the plant species might be crucial (Hegnauer, 2001). However, various plants can also react differently to a specific insect herbivore (Arimura et al., 2005; Wu and Baldwin, 2010; Hogenhout and Bos, 2011; Sanchez-Arcos et al., 2016). Such differential plant responses can become apparent in changes in i.e., the plant metabolome (Jansen et al., 2009; Tzin et al., 2015).

Secondary metabolites used by plants as defenses against herbivores can act directly as feeding deterrents or toxins that decrease food intake or food-utilization efficiency (Gabrys and Tjallingii, 2002; Kim et al., 2008), decrease survival (Behmer et al., 2011), or indirectly as attractants for natural enemies of herbivores (reviewed in Unsicker et al., 2009). Chemical compounds involved in direct and indirect plant defense include terpenoids, phenolics, cyanogenic glycosides, glucosinolates, and alkaloids (Dreyer et al., 1985; Avé et al., 1987; Zagrobelny et al., 2004; Unsicker et al., 2009; Mithöfer and Boland, 2012; Maag et al., 2015; Züst and Agrawal, 2016). Specialized insect herbivores have to somehow cope with the presence of defensive secondary metabolites in their host plants, and have evolved specific adaptations to enable feeding (Ehrlich and Raven, 1964; Berenbaum and Zangerl, 1998).

To evaluate the potential defensive effects of plant metabolites on insect herbivores, the identity and concentration of these substances must be measured. Targeted analyses can be used for such studies if previous knowledge suggests that a specific metabolite or metabolite class like flavonoids or glucosinolates might be important. However, in some cases previous knowledge about the chemical composition of the plants involved or compounds relevant to herbivores is not available. In these cases the untargeted investigation of the whole metabolome by the use of metabolomics, might be a good tool to reveal candidate chemicals that are the cause of different plant-insect interactions (Fiehn, 2002; Krastanov, 2010; Nakabayashi and Saito, 2013).

One especially interesting plant-insect herbivore system to study the role of plant metabolites for speciation is the interaction between the pea aphid complex (*Acyrthosiphon pisum* Harris, Homoptera, Aphididae) and its legume (Fabaceae) host plants. The pea aphid underwent a rapid diversification about 6500–9500 years ago (Peccoud et al., 2009b) that led to the development of at least 15 different sympatric host races or biotypes each specialized on one or a few legume host plants (called native host plants) on which they perform well and prefer for feeding (Peccoud et al., 2009a, 2015). Such host plant preferences lead to assortative mating and reproductive isolation among populations (Caillaud and Via, 2000; Peccoud et al., 2009a). However, all pea aphid host races can perform well on *Vicia faba*, which is considered to be the universal host plant for all pea aphid host races characterized to date. The existence of the different host races and the fact that the pea aphid genome is entirely sequenced (The International Aphid Genomics Consortium, 2010) has made the pea aphid a model for studying ecological speciation (Brisson and Stern, 2006; Peccoud and Simon, 2010) and provides the opportunity to investigate the mechanisms underlying host plant adaptation.

Pea aphids like all aphids feed on phloem sap. With their sucking mouthparts called stylets they navigate through the epidermis and mesophyll to reach the phloem. During this penetration process they pierce many plant cells, salivate into the cells and also suck tiny amounts of cell contents (Tjallingii and Esch, 1993; Martin et al., 1997). It is assumed that aphid salivary proteins are involved in the adaptation of pea aphid host races to host plants (Jaquiery et al., 2012). Recent studies with other aphid species have revealed a close relation between proteins secreted with the saliva into the plant and host plant reactions (Rodriguez and Bos, 2013; Kaloshian and Walling, 2016). While several aphid proteins were found to facilitate aphid feeding (Will et al., 2007; Bos et al., 2010; Atamian et al., 2013; Elzinga et al., 2014; Naessens et al., 2015); other aphid proteins induce defense reactions in the plant and could lead to an incompatible aphid – plant interaction (Chaudhary et al., 2014; Elzinga et al., 2014). Concerning the pea aphid host races and their adaptation to their native hosts, despite the efforts made to investigate the role of candidate saliva proteins on host plant adaptation (Jaquiery et al., 2012; Guy et al., 2016; Boulain et al., 2018; Nouhaud et al., 2018) saliva proteins important for host plant specialization are not yet known. However, it is known that legumes differ in their production of defense hormones depending on whether native or non-native pea aphid host races are feeding on plants (Sanchez-Arcos et al., 2016). These hormone differences might lead to changes in plant metabolomes, especially for compounds having a deterrent or toxic impact on aphids.

Several studies show that plant secondary compounds have detrimental effects on pea aphids (Züst and Agrawal, 2016). For example, higher levels of saponins and phenolic compounds led to a reduction of aphid population growth (Golawska et al., 2006; Golawska and Lukasik, 2009) and increased mortality (De Geyter et al., 2012). Diverse flavonoid glycosides reduced aphid fecundity (Golawska and Lukasik, 2012; Golawska et al., 2012a), while nitrogen-containing compounds caused a rejection of a potential host plant by pea aphids (Kordan et al., 2012). Although these targeted approaches revealed that some secondary metabolites affect pea aphid performance, in most of these studies just one plant species was used and often the pea aphid host race was unknown. Thus, the contribution of plant compounds to the maintenance and performance of pea aphid host races on legume plants is still largely unknown. Only one study (Hopkins et al., 2017) combined a metabolomic profiling approach with behavioral tests to understand the chemical signatures that underlie host preferences by *A. pisum*. This study investigated the metabolome of uninfested plants of different plant species belonging to the genera *Medicago* and *Trifolium*, but was limited to constitutive defense compounds or other compounds important for initial acceptance.

To pave the way for later targeted analyses in which the contribution of plant metabolites to the maintenance and performance of pea aphid host races on different legume plants could be analyzed, we applied an untargeted mass spectrometry-based metabolomic approach. Polar and semi-polar fractions of three native host plants of the pea aphid, *Medicago sativa*, *Pisum sativum*, *Trifolium pratense*, and the universal host *Vicia faba*, each infested with their native or one of two non-native aphid host races were analyzed and compared to fractions of uninfested control plants. These data made it possible to evaluate the use of metabolomics in identifying plant metabolites potentially involved in determining host race-host plant interactions in pea aphids.

## Materials and Methods

### Plant Material

Four legume plant species, *Medicago sativa* (alfalfa) cultivar (cv.) “Giulia,” *Trifolium pratense* (red clover) cv. “Dajana,” *Pisum sativum* (pea) cv. “Baccara,” and *Vicia faba* (broad bean) cv. “The Sutton,” were grown in 10 cm diameter plastic pots with a standardized soil mixture (7:20 mixture of Klasmann Tonsubstrat and Klasmann Kultursubstrat TS1, Klasmann-Deilmann GmbH, Geeste, Germany), in climate chambers at 20°C, 70 ± 10% relative humidity, and under a 16 h light/8 h dark photoperiod. The plants were watered twice a week. To have a sufficient amount of plant material for the extraction of metabolites, *M. sativa* and *T. pratense* plants were used 4 weeks after sowing, while *P. sativum* and *V. faba* were used 3 weeks after sowing.

### Aphids

Three pea aphid (*Acyrthosiphon pisum* Harris) clones, each representing one pea aphid host race, were used in the experiments: the clone L84 representing the Medicago race (here called MR), the clone T3-8V1 representing the Trifolium race (TR), and the clone Colmar representing the Pisum race (PR). Aphids were initially collected from their native host plants *T. pratense*, *M. sativa*, and *P. sativum*, respectively, and genotypically assigned to their respective host race (for detailed information see Table S1 in Peccoud et al., 2009b). All aphids were reared on 4 week old broad bean plants. To synchronize the age of the aphids for the experiments, five apterous female adults were placed on a broad bean plant and were allowed to reproduce for 48 h and were then removed from the plants. Nymphs were kept on the plants for 9 days until they reached adulthood. Then they were transferred to new plants were they reproduced. This procedure was repeated until enough synchronized young adult aphids were available for the experiment. To avoid escape of aphids, all aphid-containing plants were covered with air permeable cellophane bags (18.8 × 39 cm, Armin Zeller, Nachf. Schütz & Co, Langenthal, Switzerland), and placed in a climate chamber under the same conditions described for the plant material.

### Experimental Design

Five adult apterous female aphids of each host race were placed in magnetic clip-cages (Ø 3.5 cm), on leaves of each plant species (two leaves for *M. sativa* and *T. pratense*, one leaf for *P. sativum* and *V. faba* plants). Leaves from all four plant species enclosed in magnetic clip cages but without aphids served as controls (Supplementary Figure 1). Ten replicates of each combination were employed. All the infested and control plants were placed in climate chambers at 20°C, 70 ± 10% relative humidity, and under a 16 h light/8 h dark photoperiod. Plant material was sampled after 48 h, a period which allowed the aphids to settle and the plant to react to the aphid infestation (Sanchez-Arcos et al., 2016).

### Plant Material Sampling and Metabolite Extraction

For plant material sampling, the clip cages were carefully opened, and aphids were removed using a paintbrush. Control plants without aphids were brushed in the same way as aphid-infested plants to control for possible induction of metabolic changes due to contact with the paintbrush. Leaves enclosed in the clip cages were harvested and rapidly frozen in liquid nitrogen. Frozen samples were stored overnight in Eppendorf tubes (2 ml) at −80°C and then freeze-dried for 48 h. Dried plant material was homogenized into a fine powder by adding three stainless steel beads (3 mm Ø) in each tube and vigorous shaking for 4 min on a paint shaker (Skandex shaker SO-10m, Fast & Fluid Management, Sassenheim, The Netherlands). Portions of 10 mg dried plant material were extracted with 1 ml ice-cold extraction solution containing 80% methanol acidified with 0.1% formic acid and 0.1 μg/ml of L-(+)-α-phenylglycine (as a lock mass internal standard). Samples were immediately vortexed for 10 s and continuously sonicated in a water bath at room temperature (20°C) for 15 min at a maximum frequency of 35 kHz. After centrifugation (10 min at 4,500 g and −10°C), supernatants were filtered using 0.45 μm PTFE AcroPrep^TM^ 96-well filtration plates (Pall Corporation, Port Washington, NY, United States) and a vacuum filtration unit. All filtered plant extracts were stored at −80°C until LC-Orbitrap-MS analysis.

### Plant Extract Analysis

From each plant extract 10 μl were analyzed using a UHPLC system of the Ultimate 3000 series RSLC (Dionex, Sunnyvale, CA, United States) connected to an LTQ-Orbitrap XL mass spectrometer (Thermo Fisher Scientific, Bremen, Germany). UHPLC was performed on an Acclaim^TM^ C18 column (150 × 2.1 mm, 2.2 μm, Dionex) pre-fitted with a C-18, 3.5 μm guard column (2.1 × 10 mm, Waters, Dublin, Ireland). Separation was accomplished using a gradient of 0.1% (v/v) formic acid in water (solvent A) and 0.1% formic acid in acetonitrile (solvent B) as follows: 0–5 min isocratic 100% (v/v) A, 5–32 min gradient phase to 100% B, 32–42 min isocratic 100% B, 42–42.1 min gradient phase to 100% (v/v) A, 42.1–47 min isocratic 100% A. The flow rate was set to 300 μl min^−1^. The electrospray ionization (ESI) source parameters were set to 4.5 kV spray voltage, and 35 V capillary transfer voltage at a capillary temperature of 275°C. The samples were measured in the negative (NI) and positive (PI) ionization modes in separate runs using 30,000 m/Δm resolving power (mass range of *m*/*z* 150–2000) in the Orbitrap mass analyzer. Xcalibur^TM^ software (Thermo Fisher Scientific^TM^, Waltham, MA, United States) was used for data acquisition and visualization.

UHPLC-MS^2^ analysis of selected compounds was carried out by injecting l μl of each extract into a UHPLC system (Dionex UltiMate 3000, Thermo Fisher Scientific, Dreieich, Germany) coupled to a Q-Exactive Plus mass spectrometer (Thermo Fisher Scientific). Separation was performed on a Accucore^TM^ C18 column (2.1 × 100 mm, 2.6 μm, Thermo Fisher Scientific), using a gradient of 0.1% (v/v) formic acid in water (solvent A) and 0.1% formic acid in acetonitrile (solvent B) as follows: 0–0.2 min isocratic 100% (v/v) A, 0.2–8 min gradient phase to 100% B, 8–11 min isocratic 100% B, 11.1–12 min isocratic 100% (v/v) A. A constant flow rate of 400 μl min^−1^ was set. For the parallel reaction monitoring, selective ion scanning in the negative ionization mode was used with the following parameters: target ions [M-H]^−^ (*m/z* 1085.55 and *m/z* 605.19); resolution: 17,500; AGC target: 2 × 10^5^; maximum IT: 100 ms; sheath gas flow rate: 60; aux gas flow rate: 20; sweep gas flow rate: 5; spray voltage: 3.3 kV; capillary temperature: 360°C; S-lens RF level: 50; aux gas heater temperature: 400°C; acquisition time frame: 3.75–5.88 min.

### Data Preprocessing

Raw data files were converted to mzXML format files using the MSconvert tool (ProteoWizard 3.0x software) and uploaded to the interactive XCMS online platform (Tautenhahn et al., 2012). Parameter settings for XCMS data processing were as follows: A multigroup analysis was run in the centWave mode for feature detection (Δ *m*/*z* = 2.5 ppm, minimum peak width = 10 s, and maximum peak width = 60 s); correction of the retention time was performed with an obiwarp method (profStep = 1); and for chromatogram alignment: minfrac = 0.5, bw = 5, mzwid = 0.015, max = 100, minsamp = 1. Tables with the intensities of the detected features were obtained as output. Features that were missing in 3 or more out of the 10 samples for each treatment combination were classified as sporadic features and were discarded from the data set. This resulted in 5735 features in the negative ionization mode and 9057 features in the positive ionization mode.

### Data Analysis

For the initial colonization of plants by aphids, constitutive compounds that are uniquely characteristic of a plant species might play a crucial role. A feature was classified as unique to a certain plant species when it fulfilled the following criteria: It appeared in at least eight of ten samples of the plant species of interest, and was absent in the other plant species or appeared in not more than two out of ten samples per plant species. In the same way features were selected that appeared in two or more plant species. Thus 3132 features (NI) and 5323 features (PI) were analyzed. Unique features (1081 NI; 1013 PI) were filtered again to remove adducts or isotopes which resulted in 280 (NI) and 289 (PI) possible metabolites.

To determine how the metabolic profiles differed between uninfested control plants, principal component analyses (PCAs) were performed with the web-based tool MetaboAnalyst (Xia et al., 2009; Xia and Wishart, 2011). Due to technical reasons (limited number of features which can be processed in the program) 40% of the features that were near constant throughout the plant species based on the interquartile range were filtered out prior to analyses. Thus 3441 features out of 5735 features (NI) and 5434 features out of 9057 features (PI) were analyzed in the PCA. Features were then mean-centered and divided by the standard deviation of each feature (equivalent to auto scaling) to make them comparable. In order to support the results of the PCA and to check which compound classes contribute most to the separation of the different host plants, partial least squares discriminant analyses (PLS-DA) with unique features only were performed (Supplementary Figure 3).

To determine how metabolic profiles changed within a given host plant species after pea aphid infestation, several PCAs were performed. Since it was assumed that most features would not differ among plants of the same species infested with different aphid host races and uninfested control plants, PCA analyses were carried out on the 5% of metabolomic features that changed most in each species. To identify the 5% most differently regulated host race features, all features were compared by a non-parametric one-way ANOVA on ranks and sorted by their false discovery rate (FDR). Before PCA these 5% most differently regulated features were normalized by log-transformation and scaled by mean centering and division by the standard deviation of each feature (auto-scaling) to make them comparable.

To detect chemical compounds that might be involved in plant defense, we looked for features that were down regulated in plants when a native aphid race was feeding but up regulated when non-native aphid races were applied using the pattern hunter tool in the MetabolAnalyst tool. A Spearman rank correlation analysis was performed against given patterns. A pattern was specified as a series of numbers, where each number corresponded to the concentration levels of the features in the corresponding group. For instance, the pattern “2-1-2-2” corresponding to the groups “uninfested control plants – plants infested with the native aphid race – plants infested with non-native aphid race A – plants infested with non-adapted aphid race B” searched for features down regulated (positive correlation) or up regulated (negative correlation) exclusively by the native aphid race. To test whether intensities of selected features differed among the four treatments, one-way analyses of variance (one-way ANOVA) were performed. In case of significant differences, Tukey HSD tests were executed to reveal which groups were different from each other. These univariate analyses were conducted using R software version 3.2.0 (R Development Core Team, 2015).

Selected features were assigned to chemical groups through putative identifications by performing library mass searches allowing a mass deviation in all databases of 5 ppm, and checked for spectrum matches in METLIN, Human Metabolome Database (HMDB), MetFrag, MassBank, KEGG, and LipidMaps. Features not found in these libraries or databases were considered as “unknown.”

## Results

### Pea Aphid Host Plant Species Differ in Their Constitutive Metabolic Profiles

**FIGURE 1:**
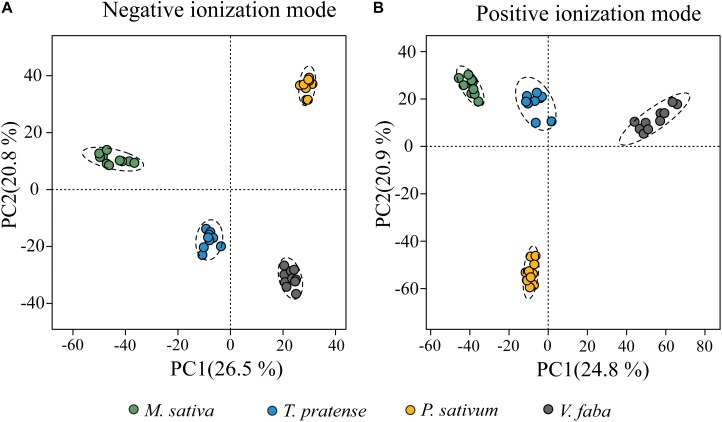
PCA plots of metabolic features of leaves of pea aphid host plant species harvested prior to infestation by aphids. Features derived from UHPLC-Orbitrap mass spectrometry of aqueous methanol were analyzed in **(A)** negative and **(B)** positive ionization modes. Colored circles represent the metabolic profiles of individual plants. Dotted ellipses represent the 95% confidence regions for each group.

To evaluate how the metabolic profiles of the four uninfested plant species, *M. sativa, T. pratense, P. sativum* and *V. faba*, differed, PCA were conducted. PCA plots showed a clear separation of all four plant species in both ionization modes (Figure 1). Biological replicates of each plant species always grouped together with small confidence intervals. The first principal components (PC1) explained 26.5 and 24.8% of the total variability for negative ionization mode (NI) and positive ionization mode (PI) datasets, respectively, whereas the second principal components (PC2) accounted for 20.8 and 20.9% (for NI and PI modes, respectively) of the total variability of the data set. For both ionization modes, the metabolic profiles of *M. sativa*, *T. pratense*, and *V. faba* were separated mainly along PC1. *P. sativum* metabolic profiles were separated from those of the other plant species along PC2.

**FIGURE 2:**
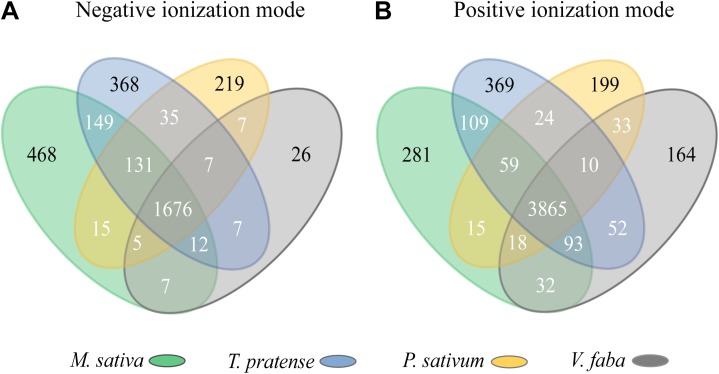
Venn diagrams depicting the metabolic features of uninfested pea aphid host plant species in **(A)** negative and **(B)** positive ionization modes. Highlighted are the features unique to each species (black numbers) and shared with others (white numbers). Colored areas enclose the values for each plant species.

To visualize the characteristic features shared among plant species, Venn diagrams were used (Figure 2). In both ionization modes, most features were shared between all four plants species (1676 out of 3132 features or 53.5% and 3865 out of 5323 features or 72.6% of all features for NI and PI, respectively), while features unique to a certain plant species were much less common. Only 34.5% (1081 features; NI) and 19% (1013 features; PI) of all features were assigned to only one plant species. *M. sativa* and *T. pratense* plants possessed a higher number of unique features in comparison to *P. sativum* and *V. faba* plants, while *V. faba* displayed the lowest number of unique features. Additionally, *M. sativa* and *T. pratense* plants shared more common features, with 149 and 109 features for NI and PI modes, respectively, than any other pair of plant species (Figure 2).

Metabolites unique to a pea aphid host plant could serve as host identification cues responsible for the acceptance or rejection of a potential host by the different host races, and might even function as defenses against non-native races. To obtain an overview of the unique compounds of each plant species, we assigned putative identifications to the unique features and organized them by chemical classes (Table 1 and Supplementary Table 1). *M. sativa* displayed the highest number of unique metabolites with 107 and 86 in NI and PI mode, respectively, followed by *T. pratense* with 103 and 83 metabolites, *P. sativum* with 57 (NI) and 53 (PI) metabolites, and *V. faba* with 13 (NI) and 67 (PI) metabolites. From all these compounds 55% (156 out of 280 compounds; NI) and 40% (115 out of 289 compounds; PI) were putatively identified. The most common class of putatively identified unique compounds among all plant species was the flavonoids. Steroidal and triterpene saponins were not only specific but also the most abundant classes in *M. sativa* (Table 1, Supplementary Table 1 and Supplementary Figure 3).

**Table 1:** Numbers of unique metabolites in uninfested *M. sativa*, *T. pratense*, *P. sativum* and *V. faba* plants and their putative chemical classification.

	Negative ionization mode		Positive ionization mode	
Plant species	Number of unique metabolites	Putative chemical class	Number of unique metabolites	Putative chemical class
*Medicago sativa*	47	Triterpene saponins	24	Triterpene saponins
	2	Steroidal saponins	14	Flavonoids
	9	Flavonoids	4	Peptides
	4	Peptides	1	Non-proteinogenic amino acid
	1	Benzoic acid ester	43	Unknowns
	1	Prostaglandin-like compound		
	1	Diterpene		
	1	Lignan glycoside		
	41	Unknowns		
	
	Σ 107		Σ 86	
	
*Trifolium pratense*	48	Flavonoids	31	Flavonoids
	6	Peptides	1	Phenolic glycoside
	2	Hydroxycinnamic acid esters	51	Unknowns
	2	Triterpene saponins		
	45	Unknowns		
	
	Σ 103		Σ 83	
	
*Pisum sativum*	22	Flavonoids	20	Flavonoids
	3	Peptides	2	Peptides
	32	Unknowns	31	Unknowns
	
	Σ 57		Σ 53	
	
*Vicia faba*	6	Flavonoids	12	Flavonoids
	1	Peptide	6	Peptides
	6	Unknowns	49	Unknowns
	
	Σ 13		Σ 67	

*Retention times, m/z values and putative chemical class of the compounds can be found in Supplementary Table 1.*

### Metabolic Profiles of Host Plant Species Are Modified Differently by the Various Pea Aphid Host Races

**FIGURE 3:**
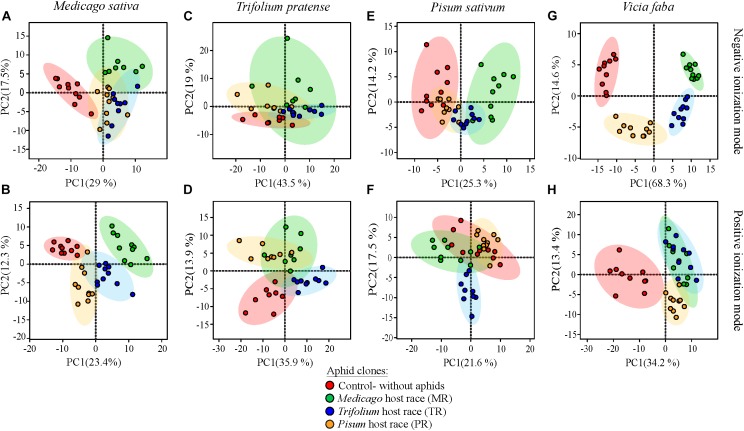
PCA plots of metabolic features of leaves of pea aphid host plant species before and after infestation by three different aphid host races. Features are derived from UHPLC-Orbitrap mass spectrometry of aqueous methanol extracts conducted in negative (top row: **A,C,E,G**) and positive (bottom row: **B,D,F,H**) ionization modes for *M. sativa* **(A,B)**, *T. pratense* **(C,D)**, *P. sativum* **(E,F)**, and *V. faba* **(G,H)**. Only the 5% of metabolomic features that changed most are plotted. Small colored circles represent individual plant metabolic profiles after infestation with the MR (green), TR (blue), or PR (yellow) host race or of uninfested control plants (red). Colored ellipses represent the 95% confidence regions for each group.

To find out how plant metabolic profiles were modified after infestation by the various pea aphid host races, PCAs were performed based on the 5% of metabolomic features that changed most among plants infested with different pea aphid host races and uninfested control plants. In general, the metabolic profiles of uninfested control plants were separated from the profiles of aphid-infested plants, and thus plant metabolomes changed significantly upon aphid infestation. The degree of separation among profiles of plants infested with different host races depended on the plant species. Whereas the metabolic profiles of *V. faba* and *M. sativa* changed substantially depending on the attacking host race, profiles of *T. pratense* and *P. sativum* changed to a smaller extent (Figure 3).

The metabolic profiles of aphid-infested *M. sativa* plants were separated from the profiles of uninfested control plants along the first principal component (Figure 3A,B). The first PC explained 29% (NI) and 23.4% (PI) of the total variability. Among the infested plants, the metabolic profiles of those infested with the native MR host race were separated from those infested with the non-native TR and PR host races mainly along the second principal component. This explained 17.5% (NI) and 12.3% (PI) of the total variability.

The metabolic profiles of aphid-infested *T. pratense* plants also separated from those of uninfested control plants, but only in the positive ionization mode (Figure 3D) and not the negative ionization mode (Figure 3C). Furthermore, those plants infested with the native TR host race were grouped apart from those of the non-native MR and PR host races, especially in the positive ionization mode (Figure 3D). Of the total variability in the metabolic profiles of *T. pratense*, 62.5% (NI) and 49.8% (PI), was explained by the first two principal components.

The proportion of variability in the metabolic profiles of *P. sativum* that could be explained by the first two principal components was slightly lower than in the other plant species, 39.5% (NI) and 39.1% (PI). In contrast to the other three plant species where metabolic profiles of uninfested plants separated well from profiles of aphid-infested plants, in *P. sativum* the metabolic profiles of uninfested plants overlapped to some extent with those of plants infested with the native PR host race (Figure 3E,F). However, in the negative ionization mode, the metabolic profiles of plants infested with the non-native MR and TR host races were separated from those of uninfested control plants along the first principal component (Figure 3E). In the positive ionization mode, the metabolic profiles of plants infested with non-native TR race separated from the metabolic profiles of the other treatments along the second principal component (Figure 3F).

A large proportion of the variability in metabolic profiles of the universal host plant *V. faba* could be explained by the first two principal components (82.9% for NI and 47.6% for PI). Metabolic profiles changed drastically between the differently treated plants, especially in the negative ionization mode. There was a clear separation between infested and uninfested plants in both ionization modes along the first principal component (Figure 3G,H). The second principal component separated MR host race-infested and uninfested plants from PR and TR host race-infested plants in the negative ionization mode (Figure 3G).

#### Some Metabolites Are Reduced by Native Pea Aphid Host Races, but Induced by Non-native Races

Pea aphid host races perform much better on their native host plants than on other species (Ferrari et al., 2006, 2008; Peccoud et al., 2009a, 2015; Schwarzkopf et al., 2013). This difference may be a consequence of the ability of each race to suppress defense signaling processes on its native plant and reduce the levels of defenses (Sanchez-Arcos et al., 2016). When feeding on a non-native host plant, on the other hand, aphid feeding may trigger signaling that leads to the induction of defenses like toxic or deterrent compounds. To test these ideas, we searched for metabolites that showed (1) significantly reduced levels only after infestation with native aphid host races, or (2) significantly increased levels only after infestation with non-native aphid host races (Table 2 and Supplementary Table 2).

**Table 2:** Numbers of compounds down-regulated only after infestation with native, but not non-native aphid host races or up-regulated only after infestation with non-native, but not native host races, and their putative chemical classification.

	Negative ionization mode		Positive ionization mode	
Plant species	Number of metabolites	Putative chemical class	Number of metabolites	Putative chemical class
*Medicago sativa*	1	Triterpene saponin	1	Triterpene saponin
	1	Isoflavonoid	1	Flavonoid
	1	Chalcone	1	Sterol
	1	Unknown	1	Jasmonate derivative
			9	Unknowns
	
	Σ 4		Σ 13	
	
*Trifolium pratense*	1	Flavonoid	1	Non-proteinogenic amino acid
	3	Unknowns	9	Unknowns
	
	Σ 4		Σ 10	
	
*Pisum sativum*	2	Unknowns	1	Flavonoid
			2	Unknowns
	
	Σ 2		Σ 3	

*Retention times, m/z values and putative formulas of the compounds can be found in Supplementary Table 2.*

**FIGURE 4:**
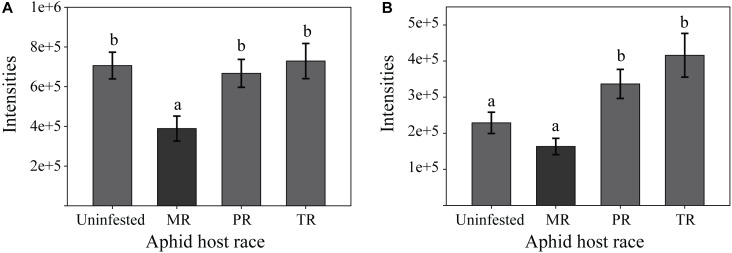
Examples of pea aphid host plant metabolites from *M. sativa* that are decreased only after infestation with the native aphid host race **(A)** or that are significantly increased only after infestation with non-native aphid host races **(B)**. **(A)** Metabolite with [M-H] ^−^ *m/z* 1085.55, was decreased only by the native MR host race. **(B)** Metabolite with [M-H]^−^ *m/z* 605.19 was induced only by the non-native PR and TR host races. Both metabolites were detected in negative ionization mode. Bars represent means ± SE. MR, Medicago race; TR, Trifolium race; PR, Pisum race; Uninfested, uninfested control plants. Different letters indicate significant differences (*P* ≤ 0.05) based on One-way ANOVA followed by Tukey HSD test. MS and MS^2^ spectra of these two compounds can be seen in Supplementary Figure 2.

*M. sativa* plants contained the highest number of metabolites that fit with these patterns, with 4 and 13 metabolites detected in the NI and PI modes, respectively, followed by *T. pratense* with 4 (NI) and 10 (PI) metabolites and *P. sativum* with 2 (NI) and 3 (PI) metabolites. A number of compounds showing these patterns could be assigned to chemical classes including several flavonoids, a triterpene saponin, a sterol and a jasmonate derivate in *M. sativa*, a flavonoid and a non-proteinogenic amino acid in *T. pratense* and a flavonoid in *P. sativum*. The abundance of two of the *M. sativa* compounds in different pea aphid treatments is illustrated in Figure 4. The levels of a putatively identified triterpene saponin (MS: with [M-H]^−^ ion at *m/z* 1085.55, [M+FA (formic acid)-H]^−^ adduct ion at *m/z* 1131.56, and [M-shikimic acid-H]^−^ ion at *m/z* 911.46; Supplementary Figures 2A,B) were decreased by aphid infestation only with the native MR host race. On the other hand, the amount of a compound, putatively classified as a glycosylated flavonoid (MS: with [M-H]^−^ ion at *m/z* 605.19, MS^2^: fragment ions at *m/z*: 577.16 and 561.20, Supplementary Figures 2C,D), was significantly increased upon infestation with the non-native TR and PR host races, but remained at similar levels as in uninfested control plants when the native MR host race infested the plant (Figure 4B).

## Discussion

### Chemical Differences Among Pea Aphid Host Plants Prior to Infestation Are Correlated With Subsequent Aphid Performance

Untargeted mass spectrometry-based analysis of the metabolites of four legume host plants of the pea aphid showed significant differences among these plants prior to aphid feeding (Figure 1). These differences might help explain the differential performance of the various pea aphid host races on specific host plants since individual metabolites could stimulate or deter feeding in disparate ways. Interestingly, the chemical complexity of the four plant species (the number of unique features found in our mass spectrometry analysis) is correlated with the performance of pea aphid host races on the various plants. *Medicago sativa*, which contained the highest number of unique chemical features (Figure 2), was previously found to be completely unsuitable for feeding by all other host races except the Medicago host race (Peccoud et al., 2009a; Schwarzkopf et al., 2013; Sanchez-Arcos et al., 2016). *Trifolium pratense*, which contained the second highest number of unique chemical features, was found to be unsuitable for other host races (Peccoud et al., 2009a), but did support some growth and reproduction of the Medicago host race (Peccoud et al., 2009a; Sanchez-Arcos et al., 2016).

The exceptional behavior of the Medicago race on *T. pratense* might be ascribed to the many chemical features shared between *M. sativa* and *T. pratense* (Figure 2), which also parallels their close phylogenetic relationship as members of the Tribe Trifolieae of the Fabaceae (Wojciechowski et al., 2004; The Legume Phylogeny Working Group et al., 2013). On the other hand, *V. faba* and *P. sativum*, both belonging to the Tribe Vicieae (Wojciechowski et al., 2004), had a much lower number of unique features, and can therefore be considered less chemically complex than *M. sativa* and *T. pratense*. This might be the reason for the good performance of all pea aphid host races on *V. faba* and their intermediate performance on *P. sativum* (Peccoud et al., 2009a; Schwarzkopf et al., 2013; Sanchez-Arcos et al., 2016).

The negative correlations between host plant chemical complexity and pea aphid performance suggest that the unique metabolites of each host might play a role in limiting aphid feeding. The most abundant class of unique chemical compounds found in *M. sativa*, and also responsible for the separation of *M. sativa* from other plant species were putatively identified as steroidal and triterpene saponins (Supplementary Figure 3). Saponins originate from the mevalonate pathway (Supplementary Figure 4), and although saponins are distributed in many plant families, they are frequently found in legumes (Vincken et al., 2007), especially the genus *Medicago*, which is well known for containing a complex mixture of triterpene saponins with a broad spectrum of biological properties (Carelli et al., 2015). Saponins are reported to be defenses against aphids (De Geyter et al., 2012; Golawska et al., 2012b, 2014) as well as other insect herbivores (De Geyter et al., 2007; Chaieb, 2010), pathogens (Oleszek et al., 1990; Abbruscato et al., 2014), and nematodes (D’Addabbo et al., 2011). Saponins exhibit strong detergent properties, and can interact with membranes disturbing permeability and leading to cell death and necrosis (Wink, 2008).

The most frequent class of unique chemical compounds putatively identified in the other pea aphid host plants studied was the flavonoids. They are synthesized by the phenylpropanoid pathway (Supplementary Figure 4) and are widely distributed in all plants (Iwashina, 2000), but flavonoids, particularly isoflavonoids, are especially abundant in legumes (Velázques et al., 2010; Wink, 2013). Flavonoids can be deterrent or toxic for insects (Brignolas et al., 1998; Widstrom and Snook, 2001; Haribal and Feeny, 2003) including phloem feeders such as aphids (Montgomery and Arn, 1974; Lattanzio et al., 2000), and also have activity against microbes (Grayer and Harborne, 1994; Rauha et al., 2000). Interestingly, in a previous metabolomics investigation of host plant choice in pea aphids, L-phenylalanine and L-tyrosine were found to be associated with differential acceptability of species of *Medicago* and *Trifolium* by the Medicago and Trifolium host races (Hopkins et al., 2017). Since L-phenylalanine is one of the major precursors of flavonoids, its correlation with pea aphid host choice may be due to the flavonoids formed from this amino acid. Thus untargeted chemical analysis of pea aphid host plants has revealed several candidate groups of compounds that may play a role in host race specificity. Future studies on these compounds are warranted to determine how they are involved in defining pea aphid-host plant interactions.

### Host Plant Compounds Induced or Suppressed by Pea Aphid Host Races May Influence Their Performance

Pea aphids may be affected not only by the constitutive chemistry of their host plants, but also by the changes induced upon aphid feeding. Thus in this study we compared the metabolic composition of host plants after feeding by the different host races with the composition of uninfested plants. Our results showed that host plant metabolomes were indeed modified by aphid feeding with modification occurring in a host race-dependent manner. The metabolic profiles of *M. sativa* and *T. pratense* plants after aphid infestation were consistently different from those of their respective uninfested control plants, and the metabolic profiles measured after feeding by the Medicago and Trifolium native host races were different from those after feeding by non-native host races (Figure 3A–D). In contrast, the metabolic profile of *P. sativum* after infestation by the native Pisum host race was not different from that of the control plants, but infestation with the non-native host races generated distinct changes in metabolic profiles. These patterns of metabolic change correspond well with changes in defense hormone levels found in a previous study after pea aphid feeding (Sanchez-Arcos et al., 2016). The salicylic and jasmonate levels of *M. sativa* and *T. pratense* were substantially altered by aphid infestation. Native host races caused slight induction or reduction of hormone levels, and non-native host races caused significant increases. In *P. sativum*, the native host race did not modify defense hormone levels compared to those found in uninfested control plants.

The metabolic changes measured after aphid feeding may result from a series of aphid behaviors that occur during the establishment of phloem feeding. En route to the phloem, aphids pierce many plant cells and secrete watery saliva into these cells (Martin et al., 1997; Powell, 2005). They also secrete gelling saliva into the apoplast to form a sheath around the stylet. Interestingly, even in incompatible interactions (such as those resulting from non-native host races) when aphids are not able to feed on the sieve elements, they insert their stylets and salivate into the plant (Schwarzkopf et al., 2013). The saliva contains proteins some of which may function as herbivore associated molecular patterns that bind to plant recognition receptors leading to the activation of plant defense responses (Hogenhout and Bos, 2011). For instance, a proteinaceous elicitor from *M. persicae* saliva induced resistance against this aphid in Arabidopsis, and pre-treatment of the host plant with aphid saliva decreased aphid fecundity (De Vos and Jander, 2009). Some of the metabolic changes observed after pea aphid infestation in this study might be a direct consequence of the defense responses induced by salivary proteins.

Aphids that are able to feed on a plant, such as native host races, may prevent the induction of defense responses by secreting effector proteins into the plant (Hogenhout and Bos, 2011; Elzinga and Jander, 2013; Van Bel and Will, 2016). For example, the effector molecule C002 from the pea aphid was shown to be essential for feeding on its universal host plant *V. faba*, and silencing of the *C002* gene reduced aphid fecundity (Mutti et al., 2008). However, plants have developed mechanisms to recognize such effector molecules and consequently activate their defense. Thus the chemical changes occurring after pea aphid infestation may reflect the outcome of the plant-aphid interaction.

The specificity of host race performance on different host plants may well be due to chemical differences among plants induced by infestation. Plants commonly synthesize new chemical compounds after herbivore attack, but this phenomenon has been studied for phloem feeding insects in only a few cases (Züst and Agrawal, 2016). Thus, in this study we sought compounds that were either induced by non-native host races but not native races, or suppressed by native host races but not non-native races. Such substances might be critical in facilitating the good performance of native host races and poor performance on non-native races. Interestingly, the number of compounds that were up or down regulated in this manner was again higher in *M. sativa* and *T. pratense* than in *P. sativum* (Table 2).

One of the compounds with these characteristics, putatively classed as a triterpene saponin, was unique to *M. sativa* and down regulated only by the native MR host race (Figure 4A). Saponins in plants have been reported to play a widespread role in resistance against insect herbivores (Applebaum et al., 1969; Fields et al., 2010; Nielsen et al., 2010), including in *M. sativa* (Nozzolillo et al., 1997; Adel et al., 2000), and in resistance against aphids in particular (Soulé et al., 2000; Golawska et al., 2012b). The adverse effects of saponins against the pea aphid have been observed in several studies (Golawska, 2007; De Geyter et al., 2012). These results suggest that the down regulation of the *M. sativa* saponin by the native Medicago host race, as described above, could be responsible for the increased performance on its native host plant. At the same time, the inability of non-native host races to down regulate this saponin may contribute to their poor performance due to the potential deterrent or toxic effects of this compound.

Another compound whose levels varied in a host race-specific manner was putatively classed as a sterol. Plant sterols are essential dietary requirements for insects, which cannot produce their own *de novo*, but need sterols as structural components of cell membranes and precursors in the formation of ecdysteroids (molting and sex hormones) (Clark and Bloch, 1959; Behmer and Nes, 2003; Behmer, 2017). However, sterols can also have negative effects on insects (Behmer, 2017). For instance the sterol stigmasterol suppressed the lifetime reproductive output of pea aphids when added to an artificial diet (Bouvaine et al., 2012). The aphid *M. persicae* had a reduced reproduction and a higher mortality when feeding on tobacco plants with high levels of atypical steroids (Behmer et al., 2011). Thus the sterol identified in this study may also represent a good candidate to be tested further with different pea aphid host races to deduce its role in determining patterns of host plant specificity.

Another compound that was induced in *M. sativa* after infestation with non-native, but not native host races was putatively classified as a flavonoid (Figure 4B). Increased levels of flavonoids in legumes after pea aphid infestation were previously reported to cause detrimental effects on the pea aphid (Golawska and Lukasik, 2012). Host race-specific regulation of flavonoids also occurred in *T. pratense*. Thus flavonoids are also good candidates to explain the differential performance of pea aphid host races.

## Conclusion

An untargeted metabolomics approach allowed the detection of a number of candidate compounds that could be responsible for the disparate performance of various pea aphid host races on leguminous plant species. Some of these candidates are constitutive substances that are unique to one of the host plants. Other candidates were differentially induced or suppressed following infestation by the various host races. The most abundant chemical classes represented were saponins and flavonoids. Based on these results, untargeted metabolomics should be more widely considered as a valuable technique to discover plant metabolites of potential importance in plant interactions with herbivores and pathogens. When related plant species differ in their susceptibility or resistance, metabolomic comparison before and after enemy attack may reveal the chemical compounds responsible.

## Author Contributions

CS-A and GK conceived and designed the experiments. CS-A performed the experiments. MK measured the metabolic profiles. CS-A and GK analyzed the data. CS-A, GK, and JG interpreted the results and wrote the manuscript. All authors critically revised and consented to the final version of the manuscript.

## Conflict of Interest Statement

The authors declare that the research was conducted in the absence of any commercial or financial relationships that could be construed as a potential conflict of interest.
