# Supplementary figures and images for: Untargeted Metabolomics Approach Reveals Differences in Host Plant Chemistry Before and After Infestation With Different Pea Aphid Host Races
Source: Front Plant Sci. 2019 Feb 28;10:188. doi: 10.3389/fpls.2019.00188 (PMC6403166; doi:10.3389/fpls.2019.00188)

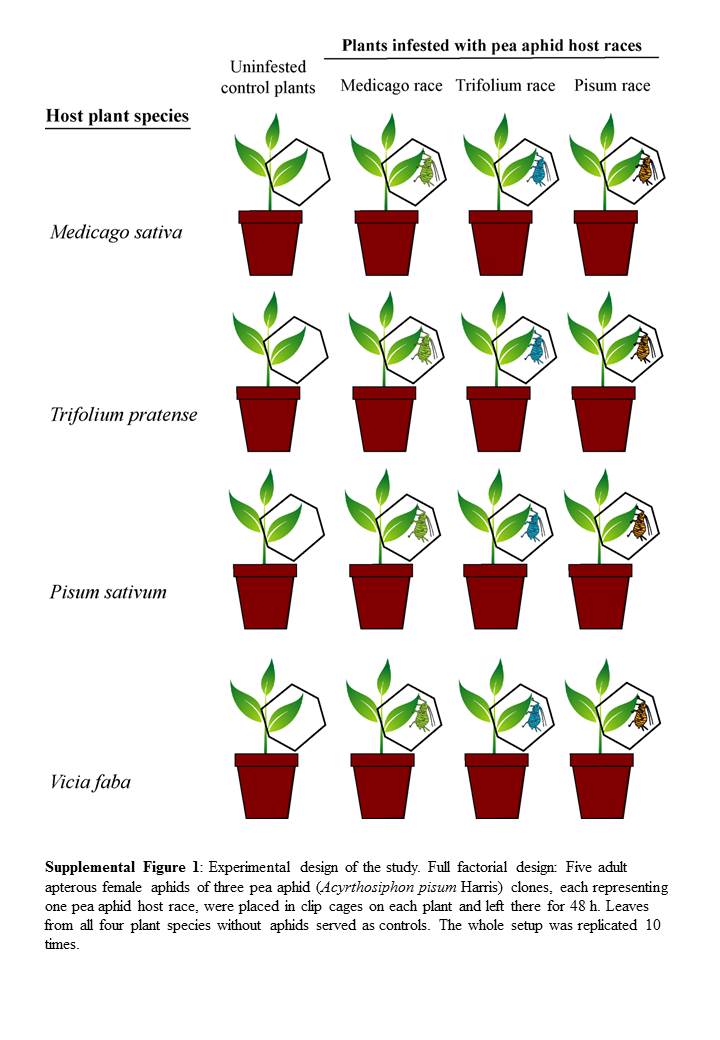

Supplement: Supplementary file 1 [file Image_1.JPEG]

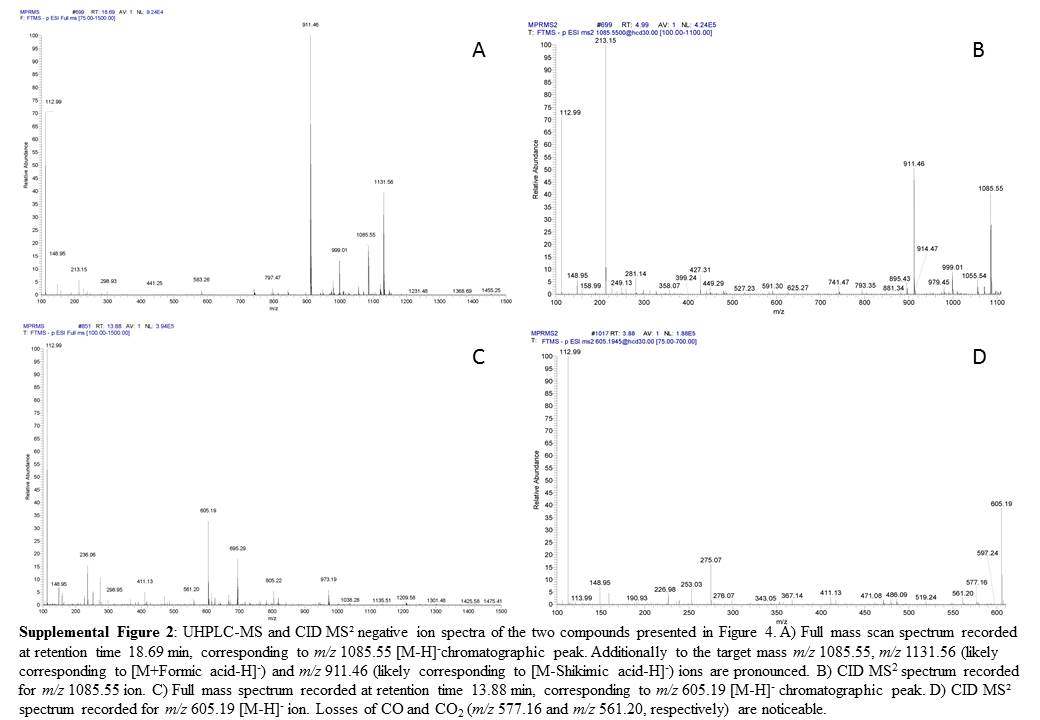

Supplement: Supplementary file 2 [file Image_2.JPEG]

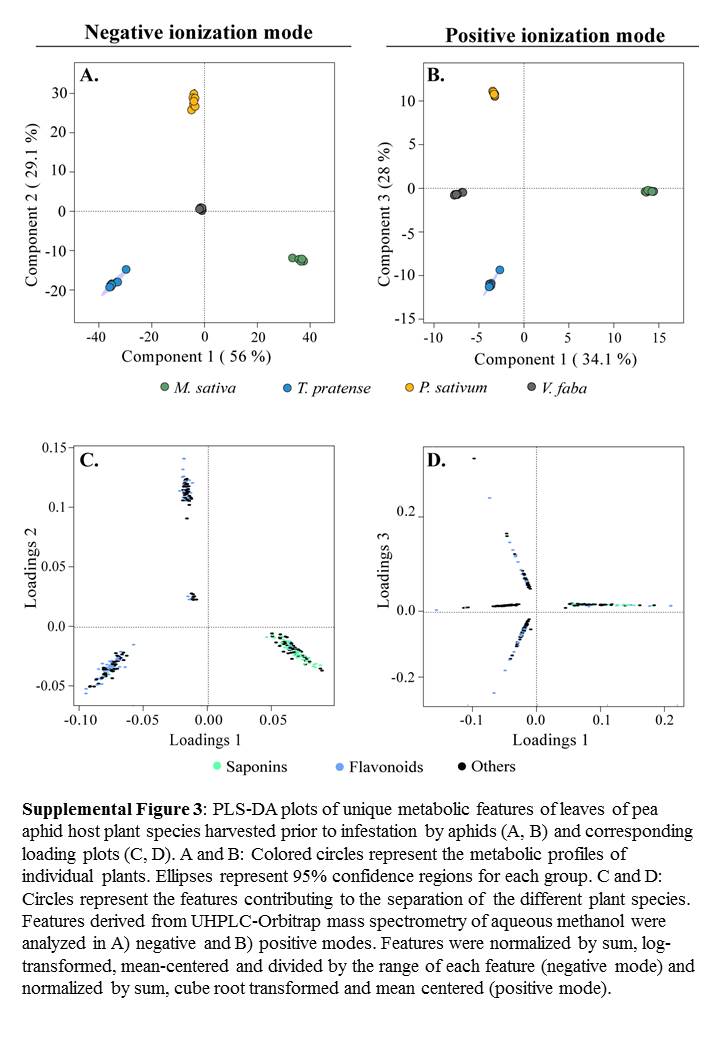

Supplement: Supplementary file 3 [file Image_3.JPEG]

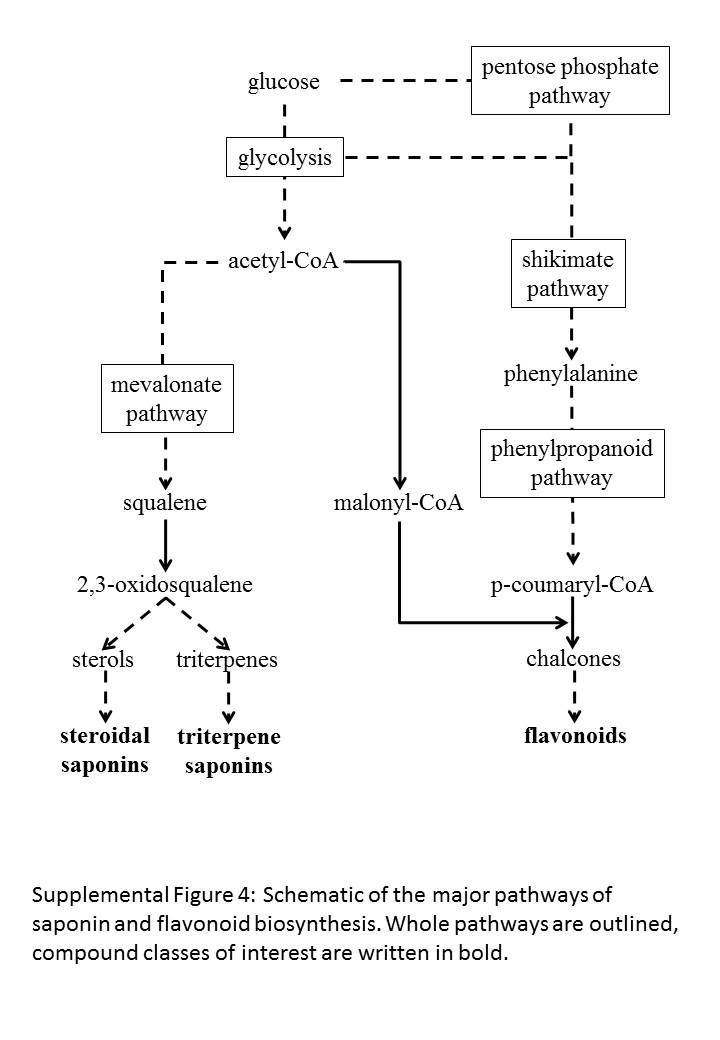

Supplement: Supplementary file 4 [file Image_4.JPEG]
